# Proposal of an Integrated Health Care Network System for Patients with Congenital Heart Defects

**DOI:** 10.5935/1678-9741.20160056

**Published:** 2016

**Authors:** Valdester Cavalcante Pinto Júnior, Rodrigo Cardoso Cavalcante, Klébia Magalhães P. Castello Branco, Candice Torres de Melo Bezerra Cavalcante, Isabel Cristina Leite Maia, Nayana Maria Gomes de Souza, Kiarelle Lourenço Penaforte, Juan Alberto Cosquillo Mejia, Waldemiro Carvalho Junior

**Affiliations:** 1 Hospital de Messejana Dr. Carlos Alberto Studart Gomes, Fortaleza, CE, Brazil

**Keywords:** Congenital Heart Disease, Delivery of Health Care, Patient Care Management

## Abstract

The perspective of the integrated health system has a network of care with multiple integration dimensions among subsystems as nuclear representation, relating the clinical aspects and governance to the representations and collective values. The normative integration aims to ensure coherence between the system of representations and values of society simultaneously with the interfaces of clinical and functional integration. It builds a bridge with governance, which allows, through their skills, management of all system components, encouraging cooperation, communication and information, in order to ensure the population under their responsibility to access excellence services, exceeding their expectations. The integration of care consists of a durable coordination of clinical practices for those who suffer from health problems in order to ensure continuity and full range of the required professional services and organizations, coordinated in time and space, in accordance with the available knowledge. It is possible to establish the type of health equipment for each level of care for patients with congenital heart diseases. This strategy intends to offer timely care in appropriate moments and places, efficiently, operating cooperatively an interdependently, with ongoing exchange of its resources. Thus, situational integration establishes the system connection with the assessment environment that proposes to carry out value judgment, guided by an objective worldview, about an intervention or any of its components, in order to objectify the decision making.

**Table:** 

**Abbreviations, acronyms & symbols**	
SUS	= Unified Health System

The perspective of the integrated health system has a network of care with multiple integration dimensions (systemic integration) among subsystems as nuclear representation, relating the clinical aspects and governance to the representations and collective values^[1]^ (Figure 1).

**Fig. 1:**
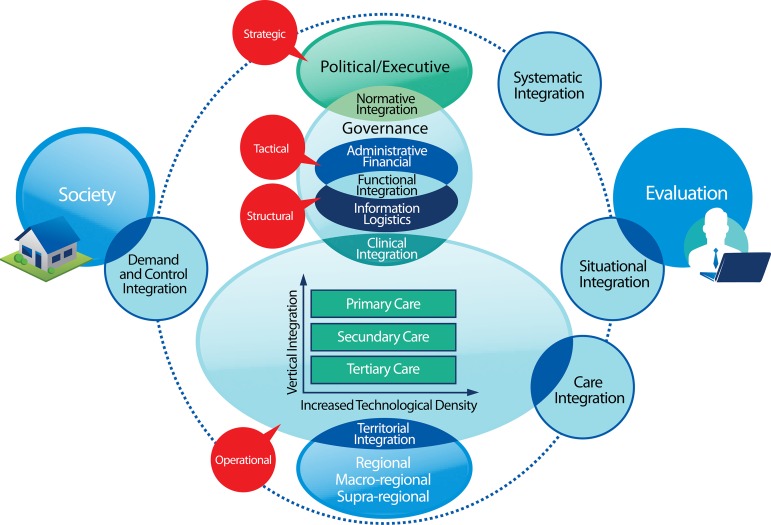
Integrated Health Care Networks for Congenital Heart Disease Patients. Adapted from Hartz & Contandriopoulos^[1]^ and Mendes^[3]^

The systemic integration is intensified in the coherence of integrational modalities in all levels (organization, territory, region, states, etc.), and we understand that a clinical project that responds to the complexity and uncertainty of health problems can result not only of relations among organizations and professionals, local relationships impacting on other levels and instances of political decision-making^[2]^.

The public policy formulation has its starting point in society demand, since it goes the political route and is legitimized by the revelation of needs and inequities. Similarly, the executive power can directly influence and create an appropriate environment for the written norms, and the more participatory, lower the risk of distortions in its deliberations. No less important than discuss the justifications and methodology of its implementation is to set goals and metrics for evaluation. Setting goals in light of social needs and political and economic reality is a negotiation exercise whose outcome must meet the principles as equity and integrity, which are in the guidelines of the Unified Health System (SUS), even not experienced in its completeness.

In this thread, the society acquires the condition and the capacity to share with other agents the political control, thus, providing the responsibility to maintain, adapt and expand its scope.

Consequently, the society starts to communicate with the system through an integration and control link.

The sensitization of the political environment moves towards influencing the decisions of the executive power, and, thus, enables continuity of the agreed actions, which should be more of a monitoring agent of the results. Even at the strategic level, the executive power, with its bureaucratic status, are entitled to format the normative content by bringing the knowledge of the SUS guidelines, conducting health actions. In this environment, the budget is allocated to all stages of the policies; however, the idealized amount is not accompanied by sufficient financial resources to solve the problems, which is explained by the lack of knowledge on the project size or the deliberate restriction of the budget to health. Anyhow, the financial failure will impact over the implementation of the policy and determine its degree of effectiveness.

The normative integration aims to ensure coherence between the system of representations and values of society simultaneously with the interfaces of clinical and functional integration^[2]^.

It builds a bridge with governance, which allows, through their skills, management of all system components, encouraging cooperation, communication and information, in order to ensure the population under their responsibility to access excellence services, exceeding their expectations.

Due to the complexity surrounding the governance, another organizational level (structural) was instituted beyond the tactical level, formed by financial and administrative environments, structural, in order to give expression to vital sectors in the development, maintenance and results of care networks. This organizational level is composed by logistics and information technology. The interface among these environments, functional integration, ensures a common coordination, guided by a system of agile and flexible information with ability to make decisions about responsibility, attributions and financial resources.

The logistics systems are technological solutions, strongly anchored in information technology, and linked to the concept of vertical integration. It consists in the realization of an effective system of reference and counter-reference of people and efficient exchange of goods and information over the health care and support systems^[3]^.

Another structural pillar in the integrated care networks in the governance environment, the information technology, had a significant development in recent decades and is a functional support, whether strategic or operational, of the providers of health care organizations. Its application extends quickness in providing information and sharing knowledge, enabling effective and agile decisions, as well as better coordination among entities. It also requires further information and capacity to handle problems related to confidential information^[4]^.

The governance interface, responsible for tactical and structural actions, and operational and clinical level, is done by clinical integration, which is based on management strategies, finance, logistics and information in an effort to provide the clinical practice of multidisciplinary skills with a view to providing comprehensive care to a given population.

The integration of care consists of a durable coordination of clinical practices for those who suffer from health problems in order to ensure continuity and full range of the required professional services and organizations, coordinated in time and space, in accordance with the available knowledge. The integration of clinical teams has as main attributes the multidisciplinary constitution of its members and its structural and participatory inclusion in the care network^[1]^.

Basic content of health care networks emerge from this definition: denote mission and common objectives; operate cooperatively and interdependently; constantly interchange their resources; are established without hierarchy among components, organize themselves in a polyarchic way in which all health care points are equally important; imply a continuum of care in primary, secondary and tertiary levels; call for a comprehensive care with promotional, preventive, curative, caregivers, rehabilitative and palliative interventions; work under the coordination of primary health care; provide timely care in appropriate moments and places, offer safe and effective services in line with the available evidence; focus on the full cycle of care to a health condition; have clear health and economic responsibilities for its population; and produce a value for its population (Figure 2).

**Fig. 2:**
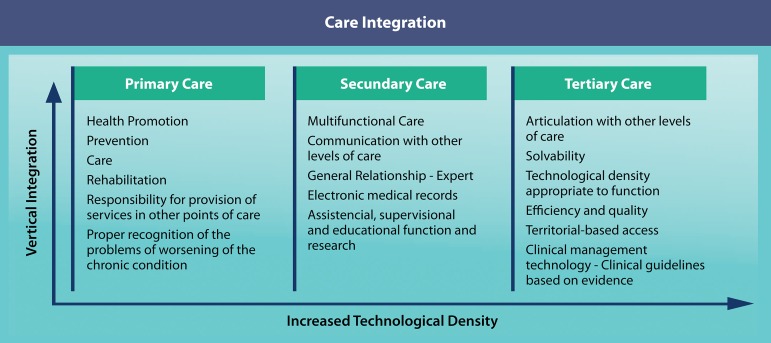
Integration of care. Attributes of primary, secondary and tertiary care levels.

The first point of care is the primary care, which can be understood as defined in the Alma Ata Conference, in 1978:

"[...] essential health care based on practical technologies, scientifically reasoned and socially acceptable, made universally accessible to individuals and families in the community through their full participation and at a cost that the community and country can maintain at every stage of their development, in the spirit of self-reliance and selfdetermination. It is the first level of contact of individuals, family and community with the national health system, whereby health care are taken as close as possible to where people live and work, and constitutes the first element of a continuing health care process"^[5]^.

The other components of health care networks are the points of secondary and tertiary care, the network nodes where they offer certain specialized services produced by a unique production function. They are distinguished by their respective technology densities, and tertiary points are technologically denser than the secondary points and, therefore, tend to be more spatially concentrated^[3]^.

It is possible to establish the type of health equipment for each level of care for patients with congenital heart diseases. This strategy intends to offer timely care in appropriate moments and places, efficiently, operating cooperatively an interdependently, with ongoing exchange of its resources (Figure 3).

**Fig. 3:**
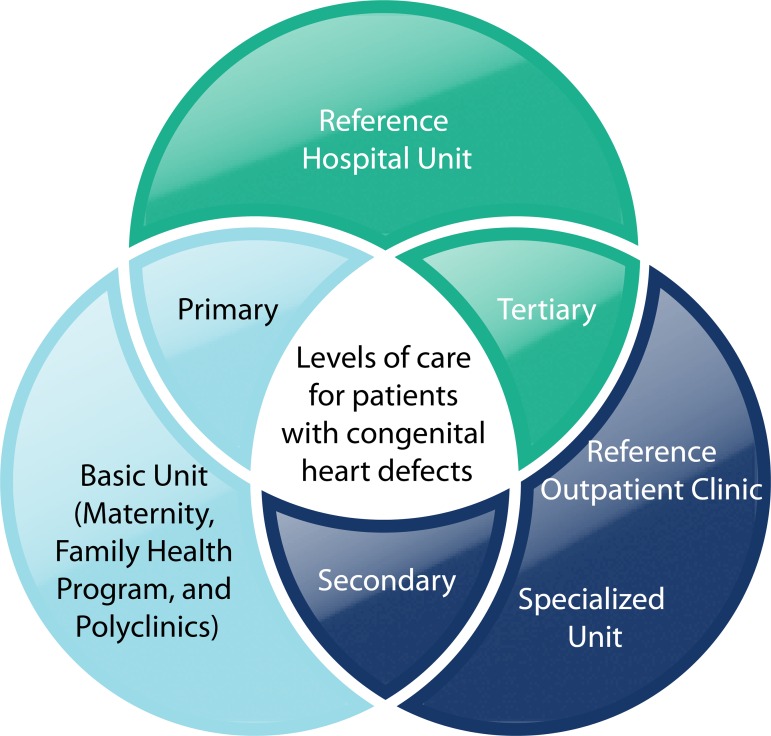
Competencies of health care levels for patients with congenital heart disease.

The integration of clinical care in the primary, secondary and tertiary care dimensions is linked to the concept of vertical integration, which refers to the combination, within the same organization or an interorganizational alliance, previously independent production units, but whose products are input from one unit to another^[6]^ (Figure 2).

By the same measure, Santana & Costa^[5]^, compiling definitions, say that vertical integration is the creation of a single management entity of two or more entities that provide services in levels of care in order to improve the overall health status of a population in a certain geo-demographic regional context. For the WHO^[7]^, vertical integration considers the aggregation of inputs, provision and service management related to the prevention, promotion, diagnosis, treatment and rehabilitation of health. It is a synonymous term with the services related to access, quality, user satisfaction and efficiency.

The motivating factors to overcome the fragmentation of health care systems are the lower transaction costs in the system and increased productivity for optimal use of common resources.

In customer perceptiveness asymmetrically informed in the face of supply agent in a disease situation, there is no perception and consecutively the capacity of decision to opt for health care consumption that offers varying levels of care. According to Costa^[8]^, the division between primary and secondary health care essentially corresponds to a preferred provider, since the perception of the consumer is focused on health care, unaware if it suffers from a problem of 'primary' or 'secondary' nature.

In this way, you can determine for each unit providing clinical care in the various levels of care, which services are made available to the user, observing concepts of vertical integration (Figure 4).

**Fig. 4:**
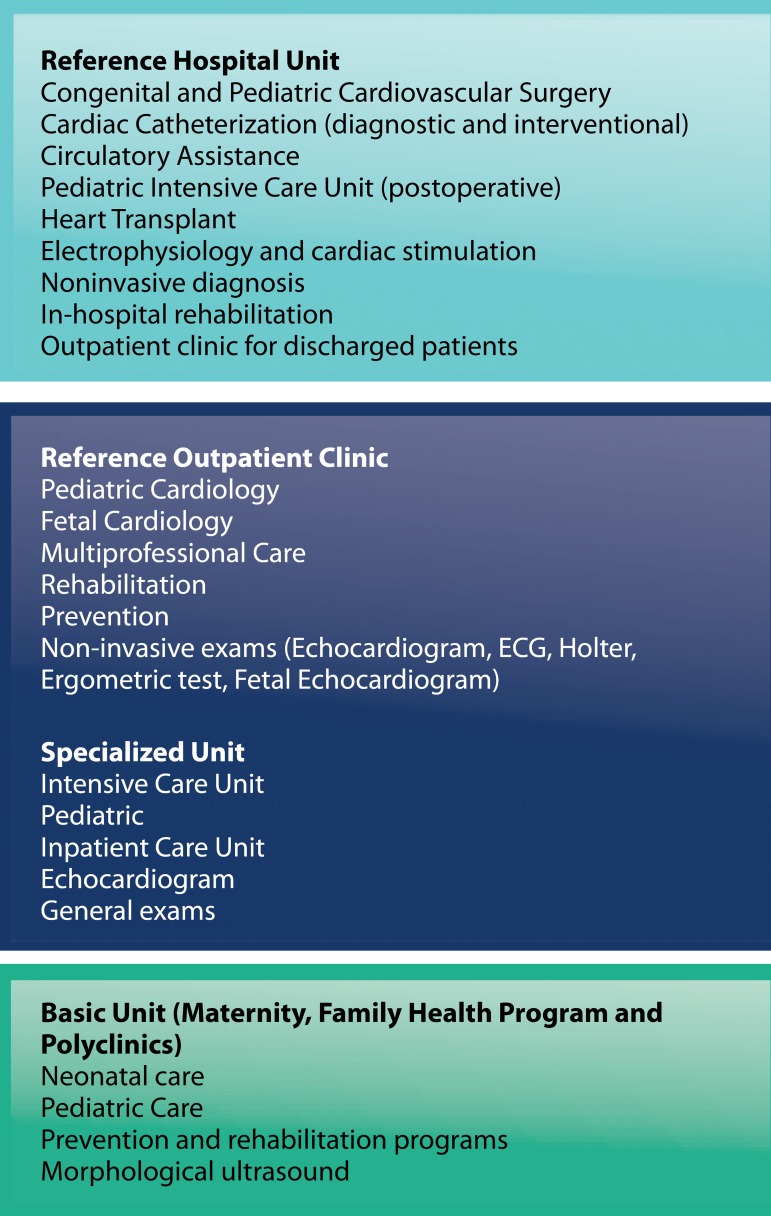
Competencies of clinical care units, related to care for patients with congenital heart disease.

In this context, territorial integration emerges, which enables the system to establish the health needs of a specific population, under its responsibility, according to the risks, and implement and evaluate sanitary interventions for this population and to provide care for people in the context of their culture and their preferences^[9]^.

Planning processes, organization, management and financing of health policy must be based on knowledge of the regional reality historically constituted and expressed in updated indicators of demographic, socioeconomic, political, epidemiological and sanitary nature, in other words, specific spaces and population. Such information and indicators should be organized and articulated in models to establish causal relationships able to guide and support the action of the State in effective policies for intervention in reality^[10]^.

The knowledge of the regional reality depends on the recognition of professional and equipment deficits able to offer specialized care to specific population segment. Therefore, it is priority to establish a formal system of allocation of resources with appropriate geographical distribution of health facilities, human resources and programs so that professional activities cover the entire spectrum of comprehensive, primary, secondary, tertiary and long-term cares, with all agreements, connections and reference needed, mechanisms established to integrate various levels and institutions in a coherent and capable group to meet all patients' needs, within a defined population-based scenario^[11]^.

In conclusion, situational integration establishes the system connection with the assessment environment that proposes to carry out value judgment, guided by an objective worldview, about an intervention or any of its components, in order to objectify the decision making. Agents must be willing to reevaluate their logics, interests and specific cultures to accept the proposed objectives, the method of work in pursuit of common goals, more ambitious than the sectored and welfare ones^[12]^.

**Table:** 

**Authors' roles & responsibilities**	
VCPJ	Conception and design; manuscript writing or critical review of its content; final approval of the manuscript
RCC	Conception and design; manuscript writing or critical review of its content; final approval of the manuscript
KMPCB	Conception and design; manuscript writing or critical review of its content; final approval of the manuscript
CTMB	Conception and design; manuscript writing or critical review of its content; final approval of the manuscript
ICLM	Conception and design; manuscript writing or critical review of its content; final approval of the manuscript
NMGS	Conception and design; manuscript writing or critical review of its content; final approval of the manuscript
KLP	Conception and design; manuscript writing or critical review of its content; final approval of the manuscript
JACM	Conception and design; manuscript writing or critical review of its content; final approval of the manuscript
WCJ	Conception and design; manuscript writing or critical review of its content; final approval of the manuscript
